# Decompression of the suprascapular nerve at the suprascapular notch under combined arthroscopic and ultrasound guidance

**DOI:** 10.1038/s41598-021-98463-1

**Published:** 2021-09-23

**Authors:** Clément Prenaud, Jeanne Loubeyre, Marc Soubeyrand

**Affiliations:** 1 Department of Orthopaedic Surgery, Public Assistance Hospital of Paris, 44 rue des Vinaigriers, 75010 Paris, France; 2 Department of Orthopaedic Surgery, Clinique Saint Jean l’Ermitage, 272 Av Marc Jacquet, 77000 Melun, France

**Keywords:** Musculoskeletal system, Nervous system, Medical research

## Abstract

Decompression of the suprascapular nerve (SSNe) at the suprascapular notch (SSNo) is usually performed with an arthroscopic procedure. This technique is well described but locating the nerve is complex because it is deeply buried and surrounded by soft tissue. We propose to combine ultrasound and arthroscopy (US-arthroscopy) to facilitate nerve localization, exposure and release. The main objective of this study was to assess the feasibility of this technique. This is an experimental, cadaveric study, carried out on ten shoulders. The first step of our technique is to locate the SSNo using an ultrasound scanner. Then an arthroscope is introduced under ultrasound control to the SSNo. A second portal is then created to dissect the pedicle and perform the ligament release. Ultrasound identification of the SSNo, endoscopic dissection and decompression of the nerve were achieved in 100% of cases. Ultrasound identification of the SSNo took an average of 3 min (± 4) while dissection and endoscopic release time took an average of 8 min (± 5). Ultrasound is an extremely powerful tool for non-invasive localization of nerves through soft tissues, but it is limited by the fact that tissue visualization is limited to the ultrasound slice plane, which is two-dimensional. On the other hand, arthroscopy (extra-articular) allows three-dimensional control of the surgical steps performed, but the locating of the nerve involves significant tissue detachment and a risk of damaging the nerve with the dissection. The combination of the two (US-arthroscopy) offers the possibility of combining the advantages of both techniques.

## Introduction

**Figure 1:**
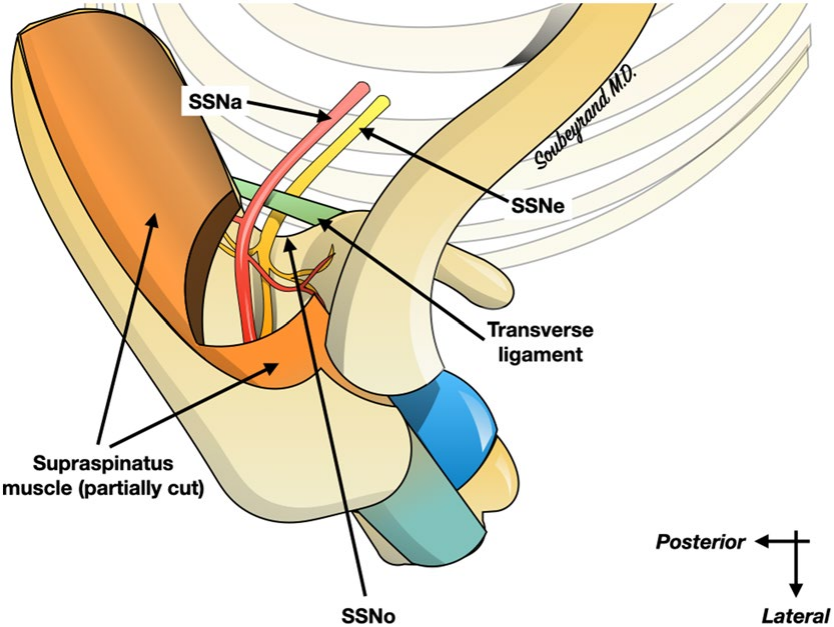
Anatomy of the suprascapular nerve (SSNe) passing through the suprascapular notch (SSNo), under the superior transverse scapular ligament, accompanied by the artery (SSA). The SSNe supplies the supraspinatus muscle as well as the infraspinatus muscle (not shown on the illustration).

Compression of the suprascapular nerve (SSNe) at the scapula is an uncommon and often misdiagnosed tunnel syndrome of the upper limb. It causes chronic scapulalgia and muscle weakness^1^. There are two potential compression zones, one at the suprascapular notch (SSNo) and the other at the spinoglenoid notch. At the level of the SSNo, the SSNe passes through the SSNo below the superior transverse scapular ligament (TL) and is accompanied by the suprascapular artery (SSA) which passes above the ligament (Fig. 1). The etiologies of SSNe compression are multiple, with different mechanisms depending on the location of the compression^2–6^. This is more frequently found at the SSNo than at the inferior scapular notch. Initially described by Thomas in 1936 and popularized by Thompson and Kopell in the late 1950s^7,8^, this compression is believed to be responsible for 2% of chronic shoulder pain^9^.

The release of the nerve at the SSNo is traditionally performed under arthroscopy or with an open surgical technique^10,11^. The arthroscopic technique is well described and is currently the reference surgery^12–14^. However, locating and releasing the SSNe is quite complex because it is deeply buried and surrounded by soft tissue. It is therefore possible to get lost in the soft tissue and never find the nerve. Even worse, during the nerve localization phase it is necessary to resect the soft tissue to create the workspace and if the operator is disoriented, he may accidentally injure the SSNe with the shaver or radiofrequency probe.

Ultrasonography (US) is a tool that makes it easy to locate the SSNo (and therefore the SSNe that passes through it) in a non-invasive way^14,15^. For example, the technique of SSNe block under ultrasound guidance is well described by the anesthesiologists^16^. We therefore propose to combine US for nerve localization and arthroscopy to control nerve dissection and TL section (US-arthroscopy) in order to achieve SSNe release at the SSNo. The main objective of this work was to evaluate the feasibility of this technique.

Our hypothesis is that the combination of arthroscopy and ultrasonography, US-arthroscopy, is anatomically and technically feasible in releasing the SSNe at the SSNo.

## Methods

This is an experimental, cadaveric study, conducted between November 2018 and March 2019, carried out on ten shoulders. We worked on cadavers coming from the Fer à Moulin Institute in Paris. We have obtained the authorization of the scientific director to carry out this study.

The first step of our technique is to locate the SSNo using an ultrasound scanner (Toshiba, Aplio, 12–14 MHz). Then an arthroscope is introduced and guided under ultrasound control to the SSNo (as an anesthetist does with his local regional anesthesia needle). A second portal is then created to dissect the pedicle and release the superior transverse scapular ligament.

### Surgical technique

**Figure 2:**
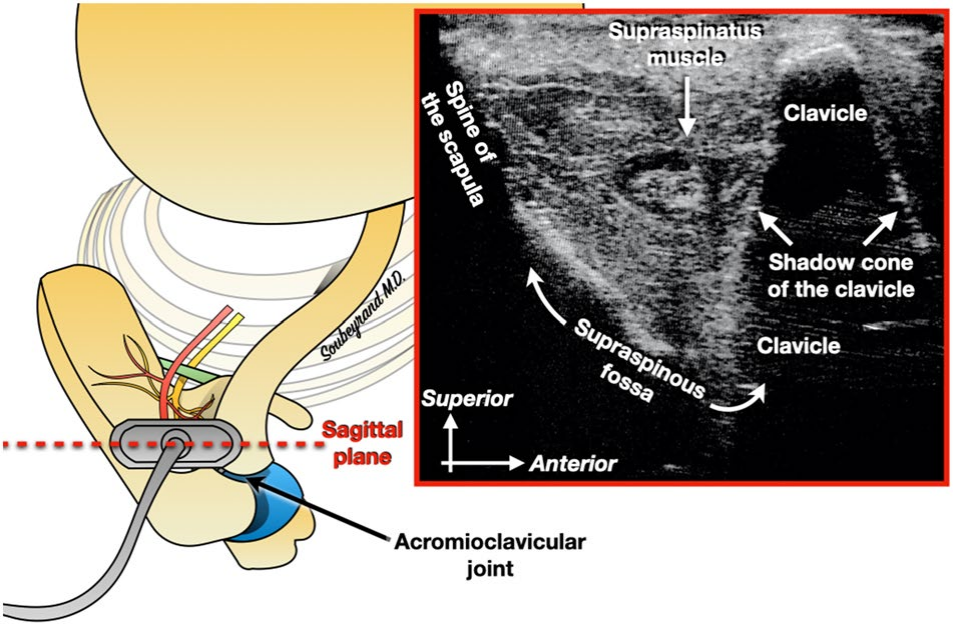
The ultrasound probe is positioned in the sagittal plane, just behind the acromioclavicular joint. The supraspinous fossa can thus be identified.

**Figure 3:**
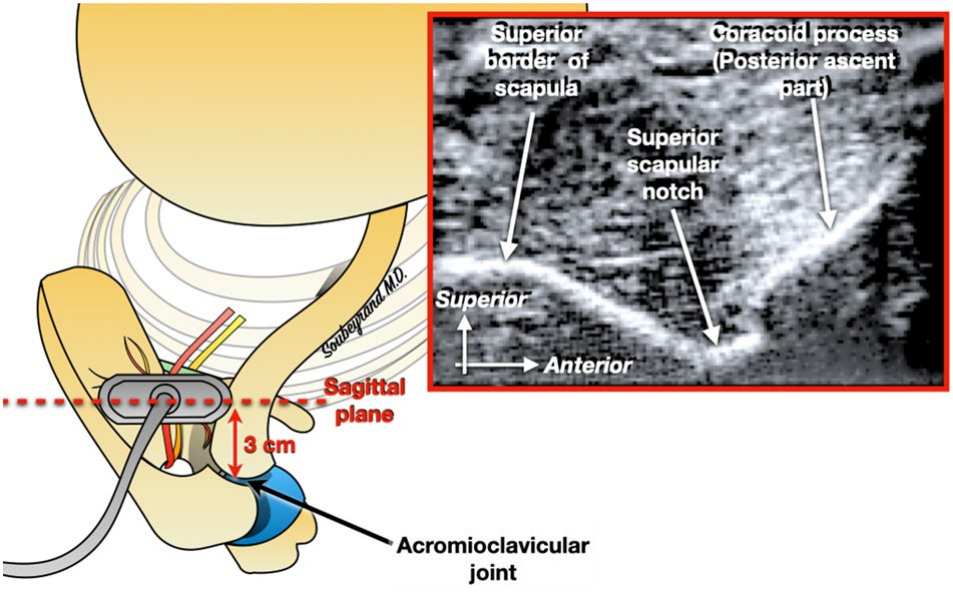
Then the probe is shifted about 3 cm medially to the acromioclavicular joint. This makes it possible to locate the superior scapular notch.

The technique is based on a first identification of the SSNo under ultrasound. First of all, the probe is positioned against the skin, in the sagittal plane, posterior to the acromioclavicular joint (Fig. 2): the supraspinous fossa is located between the spine of the scapula and the clavicle. Then the ultrasound probe is moved about 3 cm more medially which permits to locate the SSNo (Fig. 3).

**Figure 4:**
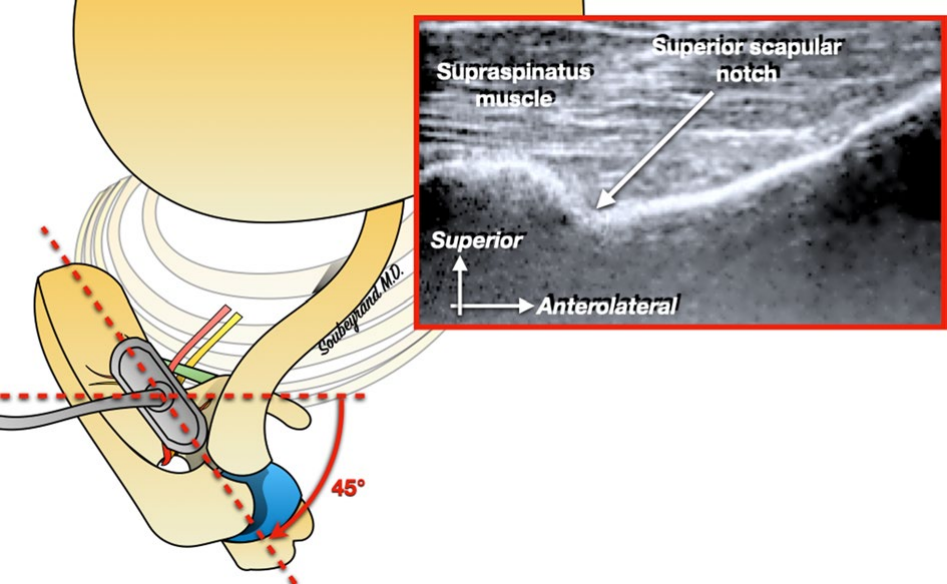
The probe is then rotated about 45°, parallel to the body of the scapula.

**Figure 5:**
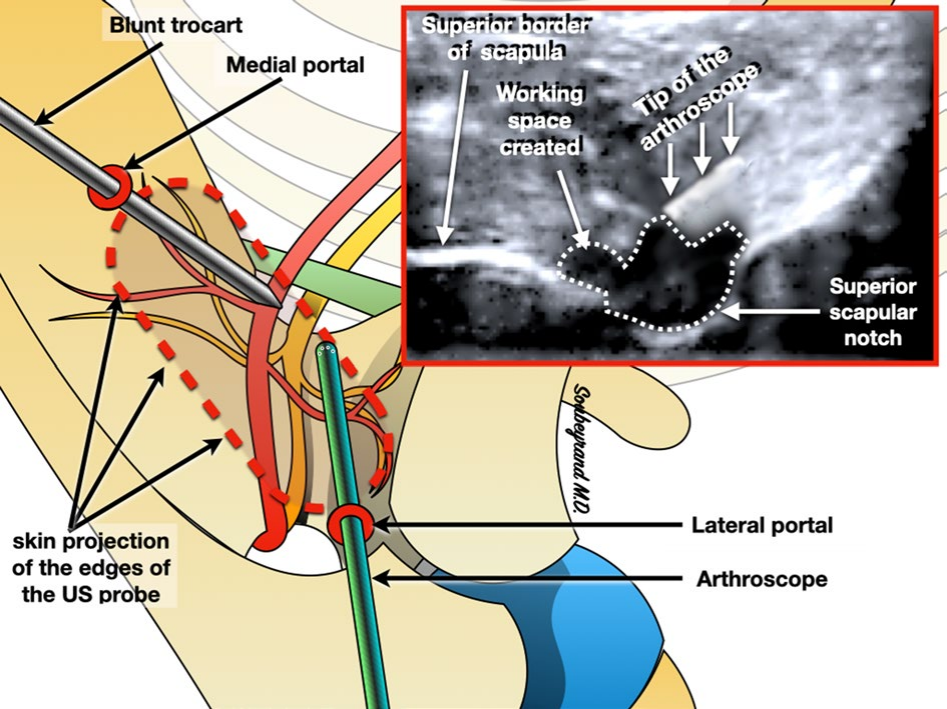
Two arthroscopic portals are created on either side of the probe (medial and lateral) in order to introduce the arthroscope and a blunt trocar. The trocar is used to create a working space around the notch: its tip is used to detach the soft tissue. The workspace gradually appears as a hypoechoic area. The arthroscope is then introduced under ultrasound guidance into the working area.

Once the notch is located, the probe is rotated by 45° and positioned parallel to the scapula: the SSNo still appears as a depression at the upper edge of the scapula (Fig. 4). With this orientation of the probe it becomes possible to create a portal on the lateral edge of the probe, through which a blunt trocart is first introduced. The latter is used to create a workspace under US guidance. Then the arthroscope is introduced, and its tip is positioned into the workspace previously created (which appears hypoechoic), directly next to the SSNo, under ultrasound guidance (Fig. 5). Once the tip of the arthroscope is correctly positioned, the arthropump is started (60 mmHg pressure). This makes it possible to expand the working space thanks to the physiological serum pressure and also to achieve hemostasis of the small vessels that may have been damaged during the creation of the working space.

**Figure 6:**
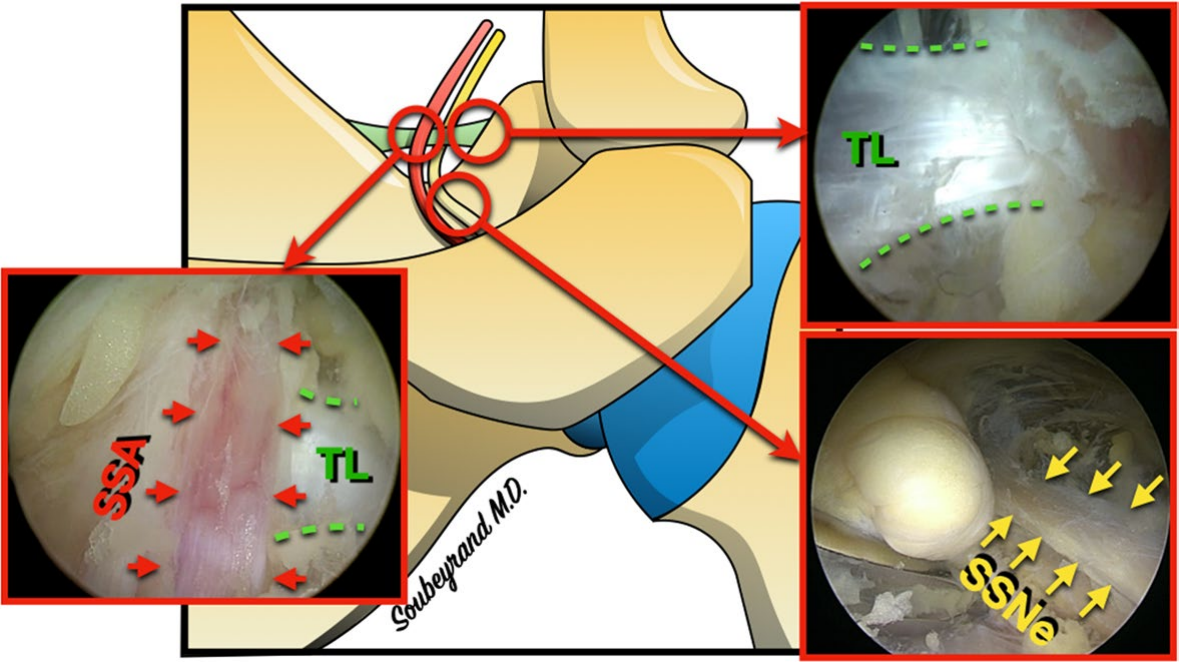
Arthroscopic aspect of the suprascapular notch (SSNo) with the suprascapular nerve (SSNe), suprascapular artery (SSA) and superior transverse scapular ligament (TL).

A second portal, medial to the US probe is then created in order to introduce the blunt trocart (Fig. 5). The latter is used to complete soft tissue dissection and retraction. Under arthroscope control the SSNo is quickly exposed as well as the SSNe, the SSA and the TL (Fig. 6).

**Figure 7:**
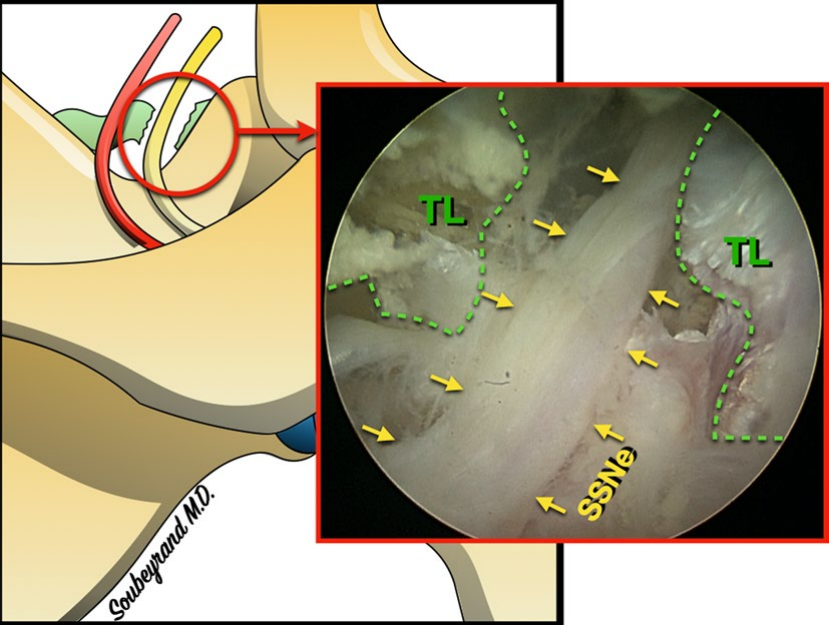
Arthroscopic appearance after section of the superior transverse scapular ligament (TL): the suprascapular nerve (SSNe) appears completely released.

Once the ligament and its pedicle have been identified, the introduction of arthroscopic scissors by the medial portal allows the ligament’s release by severing it under the control of the arthroscope (Fig. 7).

### Parameters evaluated

The time required for ultrasound identification of the SSNo as well as the duration of the arthroscopic step was measured with a chronometer. These results are reported as means and standard deviation. An open dissection was systematically carried out to ensure the effectiveness and safety of our procedure: the status of the SSNe and SSA was carefully determined, looking for possible lesions generated by the release procedure. The release of the nerve was considered satisfactory if the entire upper transverse ligament was severed.

### Ethics approval and consent to participate

This article does not contain any studies with human participants or animals performed by any of the authors. The institutional board and ethics committee of The Surgery School of the “Fer à Moulin” in Paris hereby confirms its approval for harvesting cadaveric subjects pieces. All relevant guidelines were followed for the study. We had the written informed consent from next of kin/donors for the study.

## Results

Ultrasound tracking of the SSNe, endoscopic dissection of the nerve and its satellite vessels and decompression were performed successfully in 100% of cases.

Ultrasound identification of the SSNo took an average of 3 min (± 4) while dissection and endoscopic release time took an average of 8 min (± 5).

Tree types of suprascapular notch where observed according to Rengachary^17^: types II, III and IV.

An open dissection was systematically performed looking for possible lesions generated by the release procedure. The SSNe and SSA, controlled in open dissection, were intact in 100% of cases.

## Discussion

We have shown the technical feasibility of locating the SSNo under ultrasound control and at the same time combining endoscopic release. This technique of US-arthroscopy appears to be quite simple and quick to perform.

Open release of the SSNe by the posterior approach is the traditional surgical technique, but it is not widely practiced, notably because it is dilapidating and difficult, the SSNo being deep and difficult to access^1,18^.

The arthroscopic release described by Lafosse in 2007^12^ corresponds to the reference surgical technique for the release of the nerve at the SSNo. This procedure is reliable and allows a good visualization of the suprascapular pedicle as well as a good visual control during its release. The disbonding space performed during dissection is not negligible, and the progression to the SSNo is not without risk. Indeed, to progress through the soft tissues from the subacromial space to the SSNo, the adipose and synovial interstitial tissues must be resected with the shaver or radiofrequency probe. The SSNe and SSA are contained in these tissues without a safe space around them. Thus they can appear suddenly during the progression in the soft tissues. There is therefore a risk of accidentally injuring the SSNe or SSA, the appearance of which was not anticipated. This surgery is therefore reserved for experienced and trained operators who have sufficient experience to mentalize the position of the SSNe and SSA and anticipate its appearance during dissection. It is therefore understandable that the main difficulty of this procedure is not the release of the nerve itself but rather its localization.

Ultrasound is a very powerful and non-invasive tool for imaging the inside of soft tissue. In our study, the SSNo identification was systematically carried out in a limited time in the hands of untrained operators in a reproducible manner. In practice, the use of specific modes (such as Doppler mode) present on recent models facilitates this identification procedure.

However, there is a variation of the standard deviation from the mean for the ultrasound scanning time (identification and dissection). Indeed, positioning of transducer can be challenging, and we recommend ultrasound training to develop proficiency.

Arthroscopy allows a three-dimensional visualization but at the cost of an invasive creation of the workspace as explained above (soft tissue disbonding during the progression from the subacromial space to the SSNo). By locating the SSNo non-invasively we have shown that it is possible to create a limited working space directly in contact with the SSNo in the area of interest. The rest of the procedure is then conducted under arthroscopy because the SSNe release requires precise 3D visualization of the SSNe and SSA, which US does not allow because the visualization is limited to a two-dimensional plane. The combination of the two (US-arthroscopy) makes it possible to combine the advantages of both techniques and thus limit the disbonding space. The results of our anatomical study are particularly encouraging with a complete release in 100% of cases, in a particularly limited time.

Our technique also offers the advantage of limiting the number of arthroscopic doors to two, unlike the technique described by Lafosse^12^, which requires four: a standard posterior portal, a lateral subacromial portal, an anterolateral portal and the suprascapular nerve portal.

However, our study has several limitations.

As this is a cadaveric study, in vivo transposition is a point of discussion. Indeed, the bleeding of the soft tissues during dissection is a limiting factor to the correct exposure and release of the pedicle that is not taken into account in our study.

The use of ultrasound equipment, scanning and interpretation, needs training, and the learning curve of how to use the arthroscope and ultrasound simultaneously requires time to adapt.

The performance of targeted surgery is one of the positive aspects of US-arthroscopy. However, it does not allow, during the same procedure, to associate a gesture on the inferior scapular notch.

We hypothesize that the technique combining ultrasound and arthroscopy allows less soft tissue debonding than the purely arthroscopic technique. However, we have only one group in which we have combined the two techniques and no pure arthroscopic group for comparison. However, it seems quite logical to think that avoiding the entire zone of progression between the subacromial space and the region of interest reduces the volume of tissue debonding (the size of the working area being equivalent).

As with any technique there is a learning curve that can be more or less long. In our study we did not evaluate this learning curve because our objective was primarily to determine the technical feasibility of nerve release under ultrasound. The evaluation of the learning curve will be the subject of further work.

Ultrasound is a very good imaging tool for the brachial plexus. The learning process of plexus ultrasound is considered to be rather fast^19^. At the same time, some surgeons such as Lafosse^20^ propose to release the brachial plexus under arthroscopic control in order to limit the invasiveness of the classical direct approach. However, the orientation under arthroscopy is much more complex than in the open air. In the light of this work on the suprascapular nerve, we hypothesize that arthroscopic exploration of the brachial plexus could be facilitated by the combined use of ultrasound. Of course, this has yet to be demonstrated and protocolized.

## Conclusion

Ultrasound is an extremely powerful tool for non-invasive localization of anatomical structures through soft tissues, but it remains limited to two-dimensional exploration. On the other hand, (extra-articular) arthroscopy allows a three-dimensional control of the surgical procedure. However, the localization of structures such as nerves that are embedded in the interstitial tissues (adipose, bursal) is complex, requires a soft tissue detachment sometimes important and exposes to the risk of damaging the nerve during exploration. Indeed, this exploration is performed with invasive tools such as the shaver or the radiofrequency probe, which are very effective in resecting soft tissue but can also be dangerous for the nerve.

Thus, the combination of arthroscopy and ultrasonography, US-arthroscopy, offers the possibility of combining the advantages of both techniques. This study provides evidence that the technique is anatomically and technically feasible. These positive results encourage the application and evaluation of this technique in vivo.

## Data Availability

The datasets used and/or analysed during the current study are available form the corresponding author on reasonable request.

## Abbreviations

SSNe: Suprascapular nerve
SSNo: Suprascapular notch
US-arthroscopy: Ultrasound and arthroscopy
SSA: Suprascapular artery
TL: Superior transverse scapular ligament
